# Summary of the best evidence for the prevention and management of postoperative tracheostomy complications in laryngeal cancer patients

**DOI:** 10.3389/fonc.2026.1764483

**Published:** 2026-03-26

**Authors:** Ruibin Xing, Lengjianghai Zheng, Xiaofang Chen, Shasha Qing, Qiuping Shi, Hongying Xiao

**Affiliations:** 1 Department of Otolaryngology–Head and Neck Surgery, People’s Hospital of Deyang City, Deyang, China; 2 Department of Nursing, Xi’an Jiaotong University, Xi’an, China

**Keywords:** evidence summary, evidence-based nursing, laryngeal cancer, postoperative complications, tracheostomy

## Abstract

**Introduction:**

This study aimed to identify, evaluate, and synthesize the best available evidence for the prevention and management of postoperative tracheostomy complications in patients with laryngeal cancer.

**Methods:**

The evidence-based question was structured using the PIPOST framework. Systematic searches were conducted up to February 22, 2026, across multiple databases and resources, including BMJ Best Practice, UpToDate, the International Guideline Library, Cochrane Library, PubMed, China National Knowledge Infrastructure (CNKI), and Wanfang Data. The search targeted various evidence types such as clinical decisions, guidelines, expert consensuses, evidence summaries, systematic reviews, and randomized controlled trials (RCTs). Literature screening, quality assessment, data extraction, and evidence synthesis were performed independently by two researchers, integrating professional judgment.

**Results:**

A total of 24 publications were included, comprising 5 guidelines, 8 evidence summaries, 6 expert consensus papers, 2 clinical decisions, 1 systematic review, and 2 RCTs. From these, 48 best practice recommendations were summarized and categorized into five key themes: multidisciplinary team and systematic management, artificial airway maintenance and safety, prevention and management of complications, active rehabilitation and dysphagia management, and nutritional support and prevention of malnutrition.

**Discussion:**

This work provides a comprehensive and practical summary of the best current evidence. Healthcare professionals are recommended to apply these findings in clinical practice, tailored to specific contexts and patient preferences, in order to develop systematized, proactive, and function-oriented care protocols, which are crucial for reducing complication rates and improving patient outcomes.

**Systematic Review Registration:**

http://ebn.nursing.fudan.edu.cn/home, identifier ES20258917.

## 1 Introduction

Laryngeal carcinoma is a common malignant tumor in otolaryngology–head and neck surgery that can invade the supraglottic, glottic, and subglottic regions of the laryngeal tissue, significantly affecting patients’ physiological functions and quality of life (1). Its radical treatment largely depends on surgical intervention. However, various types of laryngeal surgeries, whether partial or total laryngectomy, often require tracheostomy or permanent tracheal stoma to maintain the patient’s airway during the acute postoperative period or long-term (2). Although this measure is crucial for ensuring patient survival, it significantly alters the normal physiological structure and environment of the upper respiratory tract, causing the loss of its natural warming, humidifying, and filtering defense functions, thereby exposing patients to a range of postoperative complications (3). The reported incidence of postoperative complications in patients undergoing laryngectomy ranges from 20% to 50% (4). A prospective study of tracheostomy further revealed that the in-hospital phase is the period with the highest concentration of complications, with an incidence rate of up to 47%, which is significantly higher than 15% after discharge (5). This underscores that hospitalization is a critical window for complication prevention and management. These complications can be systematically classified as: infectious: respiratory tract infection, pneumonia, peristomal infection; mechanical: cannula obstruction, displacement, or prolapse; local tissue injuries: pharyngeal fistula, tracheoesophageal fistula, subcutaneous emphysema, and stomal skin complications (6);and functional: swallowing dysfunction, loss of vocal ability, and delayed extubation due to long-term tube dependence, all of which severely impair quality of life and recovery confidence (7). These complications are interrelated and exacerbate each other, significantly increasing the patient’s physical suffering and psychological burden, directly leading to prolonged hospital stays, sharply increasing medical costs, and becoming important independent risk factors for increased postoperative mortality (8–10). Despite its significant clinical importance, there are currently evident limitations in nursing practice and research in this field. There is still a lack of high-quality, systematic evidence-based support for comprehensive and standardized management plans for preventing complications. This fragmented knowledge base means that clinical decisions are often guided by individual experience rather than an optimized, evidence-based care pathway.

Therefore, this study aims to develop an evidence-based practice program for the prevention and management of tracheostomy-related complications after laryngeal cancer surgery by systematically searching, rigorously evaluating, and integrating the best available evidence. Methodologically, this study constitutes an evidence summary rather than a systematic review. A systematic review seeks to answer a narrowly defined PICOS question through exhaustive synthesis of primary studies. In contrast, an evidence summary identifies and synthesizes pre-appraised evidence from sources such as guidelines, systematic reviews, and expert consensuses to inform clinical decision-making on focused topics. This pragmatic, clinically oriented approach guided our study design. These findings will provide clinicians with clear and comprehensive decision-making support, with the goals of effectively reducing the incidence of complications, improving medical safety and patient outcomes, and ultimately enhancing the long-term quality of life of patients undergoing laryngeal cancer surgery. Hence, this study systematically searches for and summarizes scientific evidence on the prevention and management of tracheostomy complications after laryngeal cancer surgery, aiming to provide evidence-based guidance for clinical practice.

## 2 Materials and methods

This study is a synthesis of previously published literature and does not involve direct interaction with human subjects or personal data. The conduct of this evidence-based review, including the search, critical appraisal, and synthesis of evidence, was performed in accordance with the principles of the Declaration of Helsinki and followed established methodological standards for evidence-based healthcare research. The study has been registered at the Fudan University Center for Evidence-Based Nursing (registration number: ES20258917).

### 2.1 Establishing evidence-based questions

This study used the JBI evidence-based healthcare PIPOST tool to establish evidence-based questions (11). The first P (population) represents the target population for evidence application, namely, patients with laryngeal cancer who have undergone tracheostomy without mechanical ventilation; I (intervention) represents the intervention, which includes various measures for complication prevention, such as stoma care, airway management, nutritional management, swallowing function training, and health education; the second P (professional) represents the healthcare professionals applying the evidence, specifically clinical nurses in the ENT and head and neck surgery departments; O (outcome) represents the outcomes of application, namely, the incidence of tracheostomy-related complications, including accidental decannulation, pneumonia, infections, chest and subcutaneous emphysema, and cannula blockage; S (setting) is the site of evidence application, which is the ENT and head and neck surgery ward; T (type of evidence) refers to the type of evidence, including computerized decision support, guidelines, recommended practices, expert consensus, evidence summaries, systematic reviews, and randomized controlled trials (RCTs).

### 2.2 Search for evidence

According to the “6S” evidence pyramid model (12), computer decision systems are retrieved from top to bottom in the form of subject headings combined with free words, including BMJ Best Practice and UpToDate. Moreover, Chinese and English guideline websites and related professional association websites, including the International Guideline Library (GIN), the National Institute of Clinical Medicine Guidebook (NICE), the Scottish Intercollegiate Guideline Network (SIGN), the Canadian Medical Association Clinical Practice Guidelines Library (CMA Infobase), the Ontario Registered Nurses Professional Association (RNAO), the American Geriatric Association (AGS), the Australian JBI Evidence-Based Health Care Center Database, the Chinese Medical Journal Network, and the China Medical Pulse Guide Network, were searched. In addition, the Cochrane Library, CINAHL, PubMed, China National Knowledge Infrastructure (CNKI), Wanfang Database and other Chinese and English databases were searched for relevant studies on the management of posttracheostomy complications in laryngeal cancer patients, as were relevant websites such as the Chinese Nursing Association and the Chinese Medical Association, as well as gray literature.

**Figure 1:**
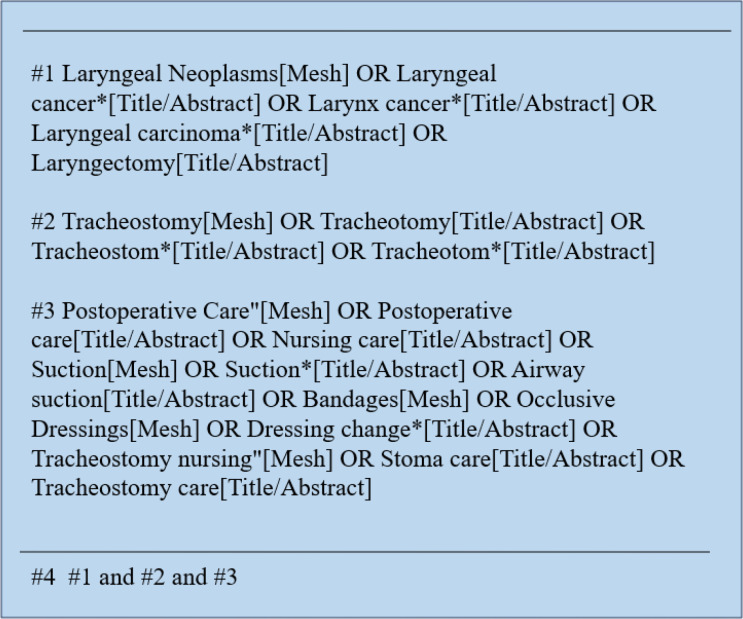
PubMed search strategy.

The search terms used in this study included “Laryngeal Neoplasms OR OR Laryngeal cancer* OR Larynx cancer* OR Laryngeal carcinoma* OR Laryngectomy”,” Tracheostomy OR Tracheotomy OR Tracheostom* OR Tracheotom*”, Postoperative Care” OR Postoperative care OR Nursing care OR Suction OR Suction* OR Airway suction OR Bandages OR Occlusive Dressings OR Dressing change* OR Tracheostomy nursing” OR Stoma care OR Tracheostomy care is the English search term. We constructed a precise search strategy based on the PICOS principle, combining medical subject headings with free words in databases such as PubMed, and limiting the type of publication (e.g., guidelines, systematic reviews). **Figure 1** provides examples of PubMed retrieval strategies. The search time limit is limited to February 22, 2026.

### 2.3 Inclusion and exclusion criteria

The inclusion criteria were as follows: studies involving patients with tracheostomy after surgery for laryngeal cancer; studies addressing nursing measures, management strategies, and interventions for preventing or managing postoperative tracheostomy complications, such as incision care, airway suctioning, dressing changes, stoma cleaning, airway humidification, nutritional management, and swallowing function training; studies involving adult patients (≥18 years) who underwent tracheostomy following laryngeal cancer surgery, regardless of their preoperative American Society of Anesthesiologists (ASA) physical status classification or the presence of comorbidities. Studies that explicitly focused on subgroups defined by specific ASA grades or comorbidities were also considered for inclusion to capture the full spectrum of clinical presentations; literature types, including computer-based decision-making, guidelines (within the last 10 years), expert consensus, evidence summaries, best practice recommendations, and systematic reviews; only the latest versions of guidelines or practice standards, are included; and studies must be in Chinese or English. The exclusion criteria were as follows: studies involving nonsurgical chemotherapy patients; duplicate publications; literature that could not answer evidence-based questions; literature that did not provide complete research results; and literature that failed quality assessment.

### 2.4 Literature screening and quality evaluation

In accordance with the 2017 Evaluation of Research and Evaluation Guidelines (AGREE II) updated by the International Collaborative Organization (13),a qualitative review of the literature was conducted, including the scope and purpose of the guidelines, participants, rigor in development, clarity of presentation, applicability, and editorial independence, with a total of 23 items and two additional overall evaluation items. Each item is rated on a scale of 1 to 7 (1 strongly disagrees and 7 strongly agrees), with higher scores indicating greater compliance with the item. The standardized score for each domain = [(actual score for each domain − lowest possible score)/(highest possible score for each domain − lowest possible score)] × 100%, and the evaluation results are divided into three levels: A level (all 6 domains ≥ 60% score, direct recommendation does not need to be modified), B grade (score ≥30% of the number of domains ≥ need to be improved and revised), and C grade (score of). <30% of the number of fields, not recommended); only Class A and B evidence was included in this study. The quality of the expert consensus was evaluated via the JBI Center for Evidence-Based Health Care Expert Consensus Evaluation Criteria (2016 edition) (14). The quality of the included systematic reviews was assessed via the JBI Australian Centre for Evidence-Based Health Care Rater Handbook (2017) (14). If the evidence comes from Up To Date, JBI evidence-based databases, etc., clinical decisions and evidence summaries are directly included and recognized as high-quality evidence. Otherwise, the original literature must be traced back and evaluated using the appropriate criteria of the JBI Centre for Evidence-Based Health Care Australia on the basis of the type of study in the original literature. Randomized controlled trials were assessed via the Cochrane risk of bias assessment tool RoB 2. The level of evidence for each recommendation was determined according to the JBI Levels of Evidence framework (2014). Under the guidance of the FAME structure, combined with the JBI recommendation strength grading principle of the evidence, the research team determined the strength of the evidence recommendation, that is, A-level recommendation (strong recommendation) and B-level recommendation (weak recommendation). This meticulous process ensures a comprehensive and meticulous evaluation of the literature.

### 2.5 Evidence extraction and integration

Two researchers trained in evidence-based care systems independently assessed the quality of the literature, and a third researcher intervened if there was a difference in opinion or decision-making difficulties. When there is overlap or conflict between the conclusions of different sources of evidence, this study follows the principles of inclusion: evidence-based evidence first, high-quality evidence first, and the latest published authoritative literature first.

## 3 Results

### 3.1 Literature search results and basic characteristics of the included studies

**Figure 2:**
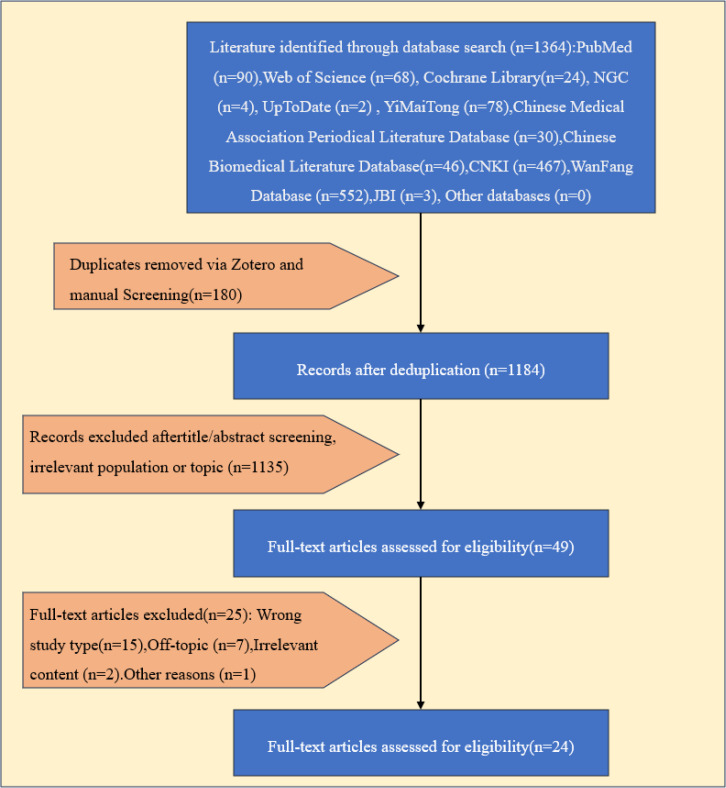
Flow chart of literature screening.

The systematic search identified 1136 records. After removing duplicates and screening titles, abstracts, and full texts, 24 articles were included for analysis. These comprised 2 UpToDate clinical decision support articles (15, 16); 5 guidelines (17–21); 8 evidence summaries (22–29); 6 expert consensuses (30–35); 1 systematic review (36)and 2 RCTs (3, 37). The literature screening process and results are shown in **Figure 2**, and the basic characteristics of the included studies are shown in **Table 1**.

**Table 1:** Characteristic of included literature (n=24).

Included literature	Publication/access year	Literature source	Literature topic	Literature type
Hyzy et al (16)	2025	UpToDate	Tracheostomy: Postoperative care, maintenance, and complications in adults	Clinical Decision
Lewin et al (15)	2024	UpToDate	Speech and swallowing rehabilitation of the patient with head and neck cancer	Clinical Decision
Mussa et al (17)	2021	AARC	AARC Clinical Practice Guideline: Management of Adult Patients with Tracheostomy in the Acute Care Setting	Guideline
Blakeman et al (18)	2022	AARC	AARC Clinical Practice Guidelines: Artificial Airway Suctioning	Guideline
NSW ACI (20)	2021	ACI	Clinical practice guide for adult tracheostomy care in acute facilities.	Guideline
McGrath et al (21)	2012	Pubmed	airway management guidelines for adult tracheostomy and laryngectomy patients	Guideline
CARM-SDRPC (19)	2023	WanFang	Evidence-based dysphagia rehabilitation guidelines.	Guideline
Chinese Expert Consensus Editorial Team (30, 31)	2023	WanFang	Chinese expert consensus on for safe adult tracheostomy tube removal	Expert Consensus
Wang et al (32)	2019	CNKI	Chinese expert consensus on Perioperative airway management in ENT surgery.	Expert Consensus
CAME-REC (33)	2023	WanFang	Chinese expert consensus on management and rehabilitation for patients with tracheostomy	Expert Consensus
CADR-NRC (34)	2024	CNKI	Chinese expert consensus on airway function rehabilitation and management for neurocritical patients with tracheostomy	Expert Consensus
CARM-RNC (35)	2021	WanFang	Chinese expert consensus on dysphagia rehabilitation care	Expert Consensus
Chang et al (27)	2023	CNKI	Evidence summary of early swallowing rehabilitation management in patients after partial laryngectomy	Evidence Summary
Luo et al (22)	2025	CNKI	Summary of the best evidence for airway humidification management in nonmechanical ventilation patients with tracheostomy after laryngectomy	Evidence Summary
Cao et al (23)	2024	CNKI	Evidence summary and translation for sputum aspiration in patients with tracheostomy	Evidence Summary
Ji et al (24)	2025	CNKI	Evidence summary for sputum aspiration in patients with tracheostomy	Evidence Summary
Feng et al (25)	2021	WanFang	Evidence summary for preventing early airway complications in nonventilated children after tracheostomy.	Evidence Summary
Liu et al (26)	2021	WanFang	Evidence summary for tracheostomy tube change procedures in long-term patients.	Evidence Summary
Guo et al (28)	2025	Pubmed	Evidence summary for Integrated Airway Management in ICU Tracheostomy Patients	Evidence Summary
Yang et al (29)	2025	Pubmed	evidence summary for perioperative enteral nutrition management in patients with laryngeal cancer	Evidence Summary
Moser et al (36)	2022	Pubmed	Prevention of Tracheostomy-Related Pressure Injury: A Systematic Review and Meta-analysis	Systematic review
Wen et al (3)	2021	Pubmed	Continuous humidification enhances postoperative recovery in laryngeal cancer tracheostomy patients	RCT
Chang et al (37)	2023	WanFang	Optimized feeding posture enhances swallowing safety and efficacy in laryngectomy rehabilitation.	RCT

AARC, American Association for Respiratory Care; NSW ACI, New South Wales Agency for Clinical Innovation.

RCT, Randomized Controlled Trial; CARM-SDRPC, Chinese Association of Rehabilitation Medicine, Swallowing Disorders Rehabilitation Professional Committee; CAME-REC, Respiratory Equipment Committee of China Association of Medical Equipment; CADR-NRC, China Association of Rehabilitation of Disabled Persons, Neurorehabilitation Committee; CARM-RNC, Rehabilitation Nursing Committee of Chinese Association of Rehabilitation Medicine.

### 3.2 Literature quality assessment

This study included a total of 5 guidelines and 2 clinical decisions. The clinical decisions were directly incorporated as high-quality evidence from authoritative databases. The quality of the relevant literature was comprehensively evaluated, and the AGREE II tool was used to assess the included guidelines. **Table 2** shows the cross-dimensional scores of the guidelines. **Table 3** lists the quality assessment results of the 6 expert consensus statements. One systematic review was included, and the detailed results of the literature quality assessment are shown in **Table 4**. For the quality assessment of 8 evidence summaries, we reviewed and evaluated the original literature on which the evidence was based; the overall quality was high, and all were included in this study. A total of 2 RCTs were included, and the evaluation results are shown in **Table 5**.

**Table 2:** Quality evaluation of included guidelines (n=5).

Included literature	Standardized percentage score by domain (%)						Recommendation (grade)	Recommendation
	Scope and purpose	People involved	Rigor of development	Clarity of presentation	Applicability	Editorial independence		
Mussa et al (17)	100	61	75	100	44	67	B	Yes
Blakeman et al (18)	97	92	82	100	22	79	B	Yes
NSW ACI (20)	100	72	69	100	69	33	B	Yes
McGrath et al (21)	100	94	83	100	81	89	A	Yes
CARM-SDRPC (19)	96	85	60	83	67	98	A	Yes

CARM-SDRPC, Chinese Association of Rehabilitation Medicine, Swallowing Disorders Rehabilitation Professional Committee.

**Table 3:** Quality evaluation results of expert consensuses (n=6).

Expert consensus	①	②	③	④	⑤	⑥
Chinese Expert Consensus Editorial Team (30)	Yes	Yes	Yes	Yes	Yes	Yes
Chinese Expert Consensus Editorial Team (31)	Yes	Yes	Yes	Yes	Yes	Yes
Wang et al (32)	Yes	Yes	Yes	Yes	Yes	Yes
CAME-REC (33)	Yes	Yes	Yes	Yes	Yes	Yes
CADR-NRC (34)	Yes	Yes	Yes	Yes	Yes	Yes
CARM-RNC (35)	Yes	Yes	Yes	Yes	Yes	Yes

CAME-REC, Respiratory Equipment Committee of China Association of Medical Equipment; CADR-NRC, China Association of Rehabilitation of Disabled Persons, Neurorehabilitation Committee; CARM-RNC, Rehabilitation Nursing Committee of Chinese Association of Rehabilitation Medicine.

**Table 4:** Quality evaluation results of systematic review (n=1).

Systematic review	①	②	③	④	⑤	⑥	⑦	⑧	⑨	⑩	⑪
Moser et al (36)	Yes	Yes	Yes	Yes	Yes	Yes	Yes	Yes	Yes	No	Yes

**Table 5:** Quality evaluation results of RCTs (n=2).

RCTs	①	②	③	④	⑤	⑥
Chang et al (37)	Some Concerns	Low Risk	Low Risk	Some Concerns	Low Risk	Some Concerns
Wen et al (3)	Some Concerns	Low Risk	Low Risk	Some Concerns	Low Risk	Some Concerns

①Bias arising from the randomization process ②Bias due to deviations from intended interventions ③Bias due to missing outcome data ④Bias in measurement of the outcome ⑤Bias in selection of the reported result ⑥Overall bias.

## 4 Summary of evidence

Researchers extracted relevant evidence from 24 included papers to form the initial draft of the evidence summary, obtaining a total of 48 pieces of evidence. The research team combined evidence with the same statements and selected conflicting evidence with high evidence levels and more recent timelines. This study classified the best evidence on the prevention and management of complications in tracheostomy patients into five major themes: multidisciplinary team and systematic management, artificial airway maintenance and safety, prevention and management of complications, active rehabilitation and management of dysphagia, nutritional support and prevention of malnutrition. By summarizing and synthesizing the best evidence presented in **Table 6**, the prevention and management of complications in tracheostomy patients can be divided into five aspects. This approach not only covers the full-cycle needs from planning to prevent complications but also fully integrates the complementary recommendations of international guidelines and local consensus, providing a scientific basis and guidance for clinical practice.

**Table 6:** Summary of evidence on the prevention and management of tracheostomy complications in postoperative laryngeal cancer patients.

Category	Evidence content	Evidence level	Recommendation level
Multidisciplinary Team and Systematic Management	1. Advocate adopting multidisciplinary tracheostomy management to reduce adverse events, achieve early decannulation, and shorten hospital stay (15, 16, 34).	Level 5	A
	2. Systematic management by a multidisciplinary team can reduce adverse events, allow for earlier extubation, and shorten hospital stays (16).	Level 1	A
	3. During interdepartmental transfer, the Identity, Situation, Background, Assessment, Recommendation (ISBAR) handover principle is used, with emphasis on the type/size of the tracheal tube, cuff pressure, swallowing function status, and the healing condition of the laryngeal cancer surgical incision (20).	Level 2	A
	4. Use standardized signage at the bedside (green for tracheostomy, red for laryngectomy) to instantly differentiate the type of patient airway, and implement standardized emergency response procedures to ensure that responders from different departments and with varying levels of experience can follow a unified, validated protocol (21).	Level 5	A
	5. Use standardized bedside labeling and emergency algorithms, and conduct multidisciplinary training (21).	Level 5	A
	6. Conduct a comprehensive preoperative assessment of the airway and overall condition. Consult the Department of Respiratory Medicine, Allergy Department, and Anesthesiology to jointly evaluate high-risk patients and develop individualized management plans (34).	Level 5	B
Artificial Airway Maintenance and Safety	7. For tracheostomy tubes with a cuff, a cuff pressure manometer should be used to regularly monitor the pressure, and the cuff pressure should be maintained at 25–30 cm H_2_O (16, 28, 31–33).	Level 5	A
	8. Continuous active airway humidification (such as using a heated humidifier) should be provided for patients after tracheostomy for laryngeal cancer to improve sputum characteristics, enhance comfort, and maintain airway patency (28).	Level 1	A
	9. For surgically implanted catheters, it is recommended to perform the first replacement 3 to 7 days after surgery; for percutaneously inserted catheters, since the stoma tract matures more slowly, it is recommended to perform the first replacement 10 to 14 days after insertion. Once the stoma has stabilized and matured, routine replacement is recommended every 30 to 90 days (16, 26).	Level 1	A
	10. Secure attachment of the tracheostomy tube flange to the peristomal dressing or neck plate with a dedicated securing device minimizes the risk of accidental tube dislodgement (16).	Level 5	A
	11. Essential components of emergency preparedness at the bedside include the immediate availability of spare tracheostomy tubes (of the same size and one size smaller) and an endotracheal tube. In the event of early tracheostomy tube displacement, the recommended approach is immediate orotracheal intubation to secure the airway (15).	Level 5	A
	12. When suctioning is indicated, strict adherence to aseptic technique is mandatory, and rough manipulation that may cause excessive patient stimulation should be avoided; additionally, routine use of humidifying fluids for titration prior to suctioning is generally unnecessary (18, 28)	Level 1	A
	13. Emergency preparedness: Clearly, specify that emergency equipment must be available at the bedside, including spare tracheostomy tubes of the same size and one size smaller, suction devices, and basic and advanced airway management equipment (20).	Level 5	A
	14. Monitoring: It is strongly recommended to use waveform carbon dioxide monitoring as early as possible to identify airway problems and confirm effective ventilation (30).	Level 5	A
	15. Airway humidification: When inhaling gas through an artificial airway, the temperature should be 34–41 °C, relative humidity 100%, and absolute humidity no less than 30 mg H_2_O/L (30).	Level 1	A
	16. Selection of humidification solution: The humidification effect of 0.45% sodium chloride solution is comparable to that of 0.9% sodium chloride solution and sterile water, but the incidence of complications is relatively lower (22, 25, 32, 34).	Level 1	A
	17. The first tracheostomy tube replacement is performed 7–10 days post-operatively, after the establishment of the tracheostomy tract (33).	Level 5	B
Prevention and Management of Complications	18. hemorrhage: Regularly(2-3h) check the stoma site, observe the nature of secretions during tracheal suctioning, and record the frequency of dressing changes to detect bleeding (16).	Level 5	A
	19. Blockage: Regularly perform endotracheal suctioning, provide humidified oxygen therapy, and establish a daily plan for cleaning or replacing the inner cannula to prevent blockage (16).	Level 5	A
	20. Tracheostomy tube displacement: By securing the flange of the tracheostomy tube to an appropriate safety strap on the tracheostomy collar around the neck, tracheostomy tube displacement can be minimized (16).	Level 5	A
	21. Infection: Oral care can effectively reduce the incidence of pneumonia and the risk of infection in patients with swallowing disorders (19, 28, 32, 34, 35).	Level 3	B
	22. Tracheo-arterial fistula: Any hemorrhage should prompt fiber optic inspection and immediate referral for definitive surgical management (20).	Level 5	A
	23. Postoperative nebulized inhalation of glucocorticoids combined with bronchodilators reduces airway inflammatory edema and maintains airway patency (32).	Level 5	B
	24. Use heated humidification via Tracheostomy High-Flow Oxygen Therapy is recommended to prevent sputum crusting and infection (22, 34).	Level 2	A
	25. Place a hydrophilic foam dressing under the tracheostomy base plate to prevent pressure injury (36).	Level 1	A
	26. Use a foam material hook-and-loop tracheostomy collar (Trach Collar) instead of traditional cloth ties (Twill Ties) for fixation (36).	Level 3	B
	27. For patients with special anatomical structures, consider using an extended or flexible tracheostomy tube (36).	Level 4	B
	28. Before and after suctioning, patients should routinely be given pure oxygen for 30–60 seconds to prevent hypoxemia (23).	Level 2	A
	29. It is recommended to regularly suction the upper respiratory tract to remove secretions above the oral cavity and tracheostomy tube to prevent aspiration-related infections (24).	Level 1	A
	30. Use aseptic technique to change the stoma dressing daily, and replace it immediately if it becomes damp. It is recommended to use precut gauze that does not shed fibers easily (25).	Level 5	A
Active Rehabilitation and Management of Dysphagia	31. Tracheostomy patients with swallowing disorders can reduce the rate of aspiration and leakage by wearing a speaking valve under the guidance of professionals, thereby helping the recovery of swallowing function (19).	Level 3	B
	32. 25, Patients with swallowing disorders after throat cancer surgery should start tongue muscle training as early as possible, which can significantly improve their swallowing function (29, 35).	Level 1	A
	33. Postural compensation improves swallowing function and reduces aspiration (19)	Level 3	B
	34. Thickeners reduce the risk of aspiration and pneumonia (19)	Level 3	B
	35. Start oral feeding with high-viscosity foods (minced meat, rice porridge), using a 30° semireclined position and supraglottic swallow technique (20, 37).	Level 2	A
	36. Assess swallowing function postoperatively, pay attention to preventing aspiration, and delay oral intake if necessary (32).	Level 5	B
	37. Perform inspiratory and expiratory resistance training (such as using a threshold pressure device), as well as active cough training, can promote extubation (33).	Level 2	B
	38. A comprehensive assessment of swallowing function must be conducted before extubation (FEES is recommended) (34).	Level 3	A
	39. A variety of tools should be used to screen and assess swallowing function, such as the repetitive saliva swallowing test, water swallow test, EAT-10 questionnaire, and volume-viscosity swallow test (35).	Level 3	A
	40. Preoperative preventive exercises and swallowing action simulation practice can reduce intraoperative injury and lay the foundation for postoperative recovery (15, 27, 29).	Level 1	B
	41. Diet adjustment: When starting to eat, it is advisable to choose solid foods that are not easily crumbled and have a uniform and smooth texture (27).	Level 5	A
	42. Adjust eating posture: Sit upright while eating, with the head slightly tilted downward toward the affected side, and swallow using the healthy side (27).	Level 5	A
	43. Adjust the bite size: It is recommended to take a bite of 5–20 ml (27).	Level 5	A
Nutritional Support and Prevention of Malnutrition	44. Screening and assessment of patients with a tracheostomy can ensure that patients considered at high nutritional risk, or who are malnourished, are identified early (15, 27, 33, 35).	Level 5	A
	45. During periods when swallowing function is unsafe, nutritional intake should be ensured through enteral nutrition (34, 35).	Level 5	A
	46. Nutritional risk screening (such as NRS 2002) should be conducted to identify patients at nutritional risk (29, 35).	Level 3	A
	47. It is recommended to provide patients with standardized dietary management early on to facilitate oral intake (27).	Level 1	A
	48. Patients should be guided on the importance of a balanced diet and diverse nutrition, as well as the benefits of quitting smoking, alcohol, and spicy foods (29).	Level 1	A

ISBAR, Identity-Situation-Background-Assessment-Recommendation;FEES, Fiberoptic Endoscopic Evaluation of Swallowing.

EAT-10, Eating Assessment Tool-10;MDT, Multidisciplinary Team; NRS 2002, Nutritional Risk Screening 2002.

## 5 Discussion

### 5.1 Standardized and multidisciplinary system for safety

The establishment of a standardized, multidisciplinary team (MDT) model in our study demonstrated significant effectiveness in preventing complications and facilitating earlier decannulation in tracheostomy patients. Our findings strongly advocate for this approach, as systematic MDT management reduces adverse events, promotes timely extubation, and shortens hospital stays (15, 16, 34). The efficacy of our model is realized through several core components: a comprehensive preoperative airway and general condition assessment involving consultations with relevant departments to formulate individualized plans (34); The ISBAR handover principle during interdepartmental transfer closes a common communication gap: critical details about tube type, cuff status, and swallowing function are systematically transmitted, reducing the risk of information loss during care transitions (21); and the implementation of unambiguous color-coded bedside signage (e.g., green for tracheostomy, red for laryngectomy) for immediate airway type recognition (21). These measures, reinforced by standardized emergency algorithms and multidisciplinary training (21), ensure a unified and validated response protocol across all clinical departments.

These results are consistent with and further substantiated by the literature. The overall benefits of MDT-based tracheostomy care in improving patient safety and decannulation outcomes are similarly reported in broader clinical settings (38, 39). The critical role of structured interdisciplinary collaboration in optimizing in-hospital management aligns with established recommendations (40), whereas the integration of simulation-based training and standardized emergency protocols further echoes evidence-based practices highlighted in prior studies (41). Moreover, our proactive strategy of weekly high-risk patient monitoring and evidence-based decannulation support is corroborated by external research demonstrating that structured MDT interventions lower adverse events and accelerate tube removal (42, 43).The weekly review allowing the team to adjust weaning plans, address emerging complications, and prevent the delays that occur when decisions are left to *ad-hoc*, single-clinician judgment. In summary, our institutional experience not only validates the effectiveness of an integrated MDT framework but also reinforces its generalizability as a replicable model for enhancing tracheostomy care from preoperative planning through emergency response.

### 5.2 Refined and proactive airway management to ensure safety and patience

The safe management of tracheostomy in laryngeal cancer patients requires a nuanced protocol that balances standardized care with individualized risk assessment. Current evidence advocates for a selective approach to tracheostomy over routine procedures to reduce complications and the risk of tumor seeding while recognizing its necessity in emergency airway obstruction (44, 45). This selective strategy should inform patient selection while maintaining rigorous technical standards. Fundamentally, proper tube management includes maintaining the cuff pressure at 25–30 cm H_2_O to prevent stenosis (16, 28, 31–33). Active airway humidification (34–41 °C, 100% humidity) serves to preserve mucosal integrity by maintaining ciliary function and mucus rheology, directly preventing the cascade of secretion thickening, crust formation, and eventual tube occlusion (46). The use of 0.45% sodium chloride for humidification is advised on the basis of its efficacy and safety profile (23, 26, 32, 34). Compared with 0.9% saline, the hypotonic solution may better approximate the osmotic characteristics of normal respiratory secretions, potentially reducing airway irritation and preserving mucociliary clearance. Tube replacement should be timed to stoma maturity 3–7 days for surgical tracheostomies, reflecting the faster tract maturation created under direct visualization, versus 10–14 days for percutaneous dilatational tracheostomies, where the tract forms more gradually and is vulnerable to false passage formation if manipulated too early (16, 27, 33). Routine replacements every 30–90 days thereafter maintain tube patency and prevent encrustation. Importantly, patients with preoperative tracheostomy often present with more advanced or aggressive disease, which may extend overall decannulation timelines due to delayed healing or adjuvant treatment needs (44). Equally important as the timing of tube changes is the manner in which these procedures, and all airway interventions, are performed. Strict aseptic technique must be maintained during any tube manipulation, incorporating nontumor principles to prevent implantation (44). Early waveform capnography provides continuous ventilation monitoring and immediate confirmation of tube position after any change or manipulation (30). Emergency preparedness requires immediate bedside access to spare tubes and an endotracheal tube for tube displacement (15, 47), with protocols adapted for total laryngectomy patients who can only be managed via their stoma (48). Decannulation decisions must account for predictors of failure, such as laryngeal collapse (49), through comprehensive laryngeal assessment. Finally, proactive management of long-term complications such as granuloma formation and edema (50) reinforces the value of standardized tube changes and vigilance monitoring. Together, these elements form an integrated safety strategy that addresses both immediate risks and long-term outcomes.

### 5.3 Predictive care and precise intervention for core complications

The management of tracheostomy complications necessitates a paradigm shift from a reactive response to a proactive strategy of predictive care and precise intervention. This approach is built upon systematic monitoring and targeted actions, a concept strongly supported by recent evidence. Predictive care for critical issues such as hemorrhage involves regular stoma checks and meticulous documentation This vigilance matters because even minor bleeding, such as small amounts of blood-tinged secretions, can be an early clinical warning sign. In some cases, such minor bleeding precedes a catastrophic tracheo-arterial fistula, making its recognition a critical opportunity for prevention (16, 20). Airway patency is maintained through a precise bundle of scheduled suctioning, heated humidification, and inner cannula care (16, 23, 34), supplemented by nebulized glucocorticoids to reduce edema (32) and pre/postsuctioning hyperoxygenation (23). The efficacy of these interventions is contingent upon staff competency, as a lack of understanding directly leads to complications such as obstruction (51), underscoring the value of specialized simulation training for emergencies (52). Infection control requires a multifocal strategy, where rigorous oral care and aseptic stoma management prevent both local infections and pneumonia (49), a critical consideration given the risk of progression to life-threatening mediastinitis in vulnerable populations (53). Concurrently, device-related complications are minimized through technological adjuvants; secure hook-and-loop collars prevent tube displacement—a common serious complication linked to factors such as obesity (54)—while hydrophilic foam dressings and customized tubes address pressure injuries and atypical anatomy (36). Foam dressings work by redistributing pressure away from the peristomal skin, while extended or flexible tubes accommodate patients with thick necks or unusual tracheal angles where standard tubes would exert excessive pressure. This entire framework is reinforced by the observed benefits of early tracheostomy in reducing risks (43, 55) and aligns with the overarching need for vigilant monitoring and multidisciplinary collaboration to standardize care pathways and solidify predictive, precise interventions as the cornerstone of safe tracheostomy management.

### 5.4 Integrated rehabilitation and nutrition

For patients who undergo tracheostomy after laryngeal cancer surgery, systematic management of swallowing dysfunction and nutritional status is crucial for recovery. Assessment begins with bedside screening tools such as the EAT-10 and volume-viscosity swallow test, but these alone are insufficient. Instrumental evaluation, particularly Fiberoptic Endoscopic Evaluation of Swallowing (FEES), provides direct visualization of laryngopharyngeal anatomy and aspiration risk, which is why it is considered the gold standard for decannulation decisions (34, 35, 56). Early identification of at-risk patients allows intervention before complications become entrenched (15, 27, 33, 35). Rehabilitation should begin preoperatively with preventive exercises and may incorporate tele-rehabilitation postoperatively (57, 58). Active strategies include professionally guided speaking valve application to reduce aspiration (19) and intensive tongue muscle training to improve pharyngeal function (29, 35), although evidence for respiratory muscle training remains limited (59). Multidimensional assessment via tools such as the MD Anderson Dysphagia Inventory (MDADI) and the Penetration-Aspiration Scale provides valuable complementary data (60, 61). For safe oral intake, a hierarchical approach combines postural adjustments, food modifications with thickeners on the basis of the physiological principles of bolus flow, and controlled bite volumes of 5–20 mL (27), all under supervised conditions. Nutritional management cannot wait for swallowing function to fully recover. Dysphagia directly compromises oral intake, and prolonged inadequate nutrition impairs wound healing and muscle strength, further delaying swallowing recovery (62, 63). For this reason, systematic nutritional screening must parallel swallowing assessment, with prompt initiation of enteral nutrition when oral intake is unsafe (34, 35). As function improves, structured transition to oral feeding, combined with patient education on balanced nutrition, supports both physical recovery and quality of life (27, 29). This integrated pathway of assessment, rehabilitation, and nutritional support effectively addresses dysphagia challenges while promoting safe decannulation and recovery.

### 5.5 Limitations and transferability

Several limitations of this evidence summary should be acknowledged. First, only two RCTs were included, reflecting the inherent challenges of conducting randomized trials in postoperative tracheostomy populations. Second, a substantial proportion of the included evidence originated from Chinese expert consensus statements. While these documents were developed through rigorous multidisciplinary processes, their international generalizability may be limited due to differences in healthcare systems, resource availability, and clinical training pathways. To mitigate this concern, we cross-referenced recommendations with established Western guidelines. High concordance was observed on core technical elements, including cuff pressure targets (25–30 cmH_2_O), active humidification parameters, and aseptic suctioning techniques—all of which are supported by consistent physiological rationale across different healthcare settings. However, several differences emerged in organizational aspects: Chinese consensus documents placed greater emphasis on multidisciplinary team structure and standardized bedside signage, whereas Western guidelines provided more detailed protocols for weaning and decannulation decision-making. These differences likely reflect variations in healthcare delivery models and professional role definitions rather than true disagreements on clinical principles. Third, restricting the search to Chinese and English may have excluded relevant evidence published in other languages. Fourth, this summary did not incorporate cost-effectiveness analyses, which are essential for informing practice in resource-constrained settings. Fifth, while this summary includes patients across a range of clinical statuses, we did not perform subgroup analyses based on preoperative ASA classification or specific comorbidities (e.g., diabetes, chronic pulmonary disease). These factors are known to significantly influence postoperative complication rates and may moderate the effectiveness of certain interventions. The applicability of our findings to patients with high ASA scores or complex comorbidities should therefore be considered with caution. Finally, although patient outcomes were a central focus, we did not systematically synthesize patient-reported outcome measures (PROMs)—such as quality of life or voice-related satisfaction—representing an important direction for future evidence synthesis.

### 5.6 Considerations for elective versus emergency tracheostomy

The majority of evidence synthesized in this review is derived from or most directly applicable to elective surgical tracheostomies, where patients are typically stable and the procedure is performed under controlled conditions. However, patients undergoing emergency tracheostomy often present with unique challenges. They may be in critical condition due to the underlying obstruction, have less time for preoperative optimization, and are at a potentially higher risk for early postoperative complications such as bleeding, subcutaneous emphysema, and tube dislodgement due to tissue edema or less mature stoma formation under urgent circumstances. While core principles of maintaining airway patency (humidification, cuff pressure management) and preventing infection are universal, the intensity of monitoring and the threshold for intervention may need to be higher in the emergency cohort. Clinicians must be vigilant for these context-specific risks. This distinction also highlights a gap in the literature, as dedicated evidence for the optimal nursing management of patients immediately following emergency tracheostomy is sparse and warrants further investigation.

## 6 Implementation considerations

**Figure 3:**
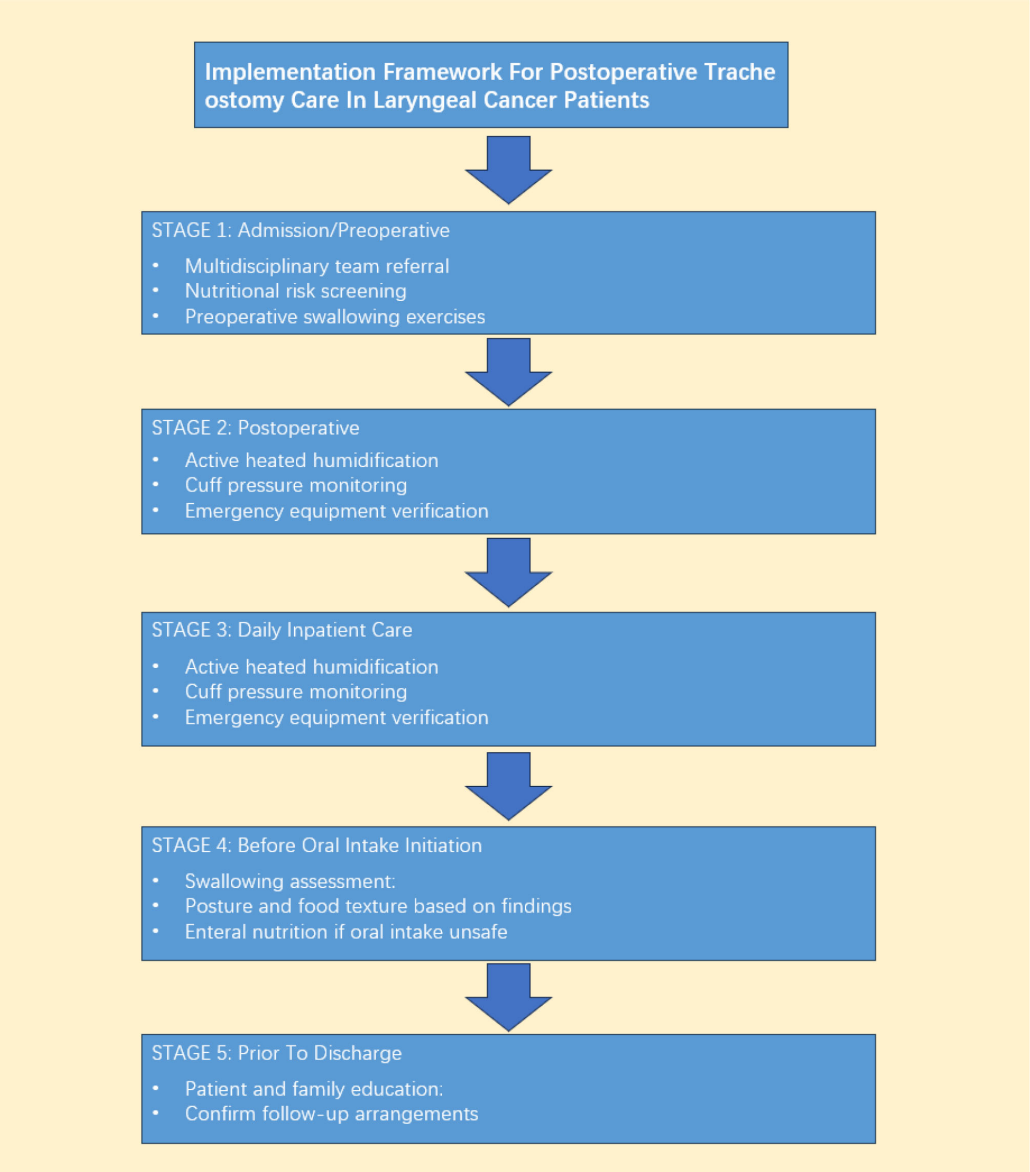
Implementation framework for postoperative tracheostomy care in laryngeal cancer patients.

To facilitate the translation of these 48 recommendations into clinical practice, we propose a time-anchored, task-oriented implementation framework organized around five critical junctures in the patient journey. At admission or preoperatively, multidisciplinary team referral and nutritional risk screening (NRS 2002) should be initiated, with preoperative swallowing exercises taught to suitable candidates. In the immediate postoperative period (first 24–48 hours), active heated humidification (34–41 °C, 100% RH) and cuff pressure monitoring (every 8 hours, target 25–30 cm H_2_O) must be established, and bedside emergency equipment—including spare tracheostomy tubes (same size and one size smaller), suction apparatus, and bag-valve mask—should be verified each shift. Daily throughout hospitalization, stoma inspection with aseptic dressing changes, assessment of secretion characteristics, and monitoring for sentinel bleeding should be performed, with suctioning delivered as clinically indicated using strict aseptic technique. Before initiating oral intake, fiberoptic endoscopic evaluation of swallowing (FEES) or validated bedside screening tools must be completed, with feeding posture and food texture modified based on assessment findings. Finally, prior to discharge, patient and family education on stoma care, emergency recognition, and suctioning techniques should be documented, and follow-up arrangements confirmed. **Figure 3** summarizes this time-anchored implementation framework.

## 7 Summary

This evidence synthesis outlines a pragmatic, phase-of-care framework for managing laryngeal cancer patients with a tracheostomy. Preoperatively, focus on multidisciplinary team involvement, nutritional risk screening, and preoperative swallowing exercises for suitable patients. In the immediate postoperative period, prioritize airway safety through active humidification, cuff pressure monitoring, and ensuring emergency equipment readiness. During daily inpatient care, emphasize prevention via aseptic stoma care, vigilant monitoring, precise suctioning, and pressure-redistributing dressings. Before oral intake, mandate formal swallowing assessment, preferably using fiberoptic endoscopic evaluation of swallowing, and tailor feeding strategies accordingly, with prompt enteral nutrition if oral intake is unsafe. Prior to decannulation and discharge, conduct comprehensive assessment of swallowing, airway patency, and cough efficacy, and provide structured patient and family education. This integrated, phase-specific approach shifts the paradigm from reactive complication management to proactive, function-preserving care, ultimately reducing complications, accelerating decannulation, and improving patient outcomes and quality of life.

## Data Availability

The original contributions presented in the study are included in the article/Supplementary Material. Further inquiries can be directed to the corresponding authors.
